# Effects of Functionalized Kraft Lignin Incorporation on Polypropylene Surface Energy and Practical Adhesion

**DOI:** 10.3390/polym14050999

**Published:** 2022-03-01

**Authors:** Manuel Patricio da Silva Bisneto, Julia Rocha Gouveia, Leonardo Dalseno Antonino, Lara Basílio Tavares, Nathalie Minako Ito, Demetrio Jackson dos Santos

**Affiliations:** 1 Nanoscience and Advanced Materials Graduate Program (PPG-Nano), Federal University of ABC (UFABC), Santo Andre 09210-580, Brazil; manuel.bisneto@ufabc.edu.br (M.P.d.S.B.); juliargouveia@gmail.com (J.R.G.); leonardoantonino@hotmail.com (L.D.A.); lara.btavares@hotmail.com (L.B.T.); nathalie.minako@gmail.com (N.M.I.); 2 Center of Engineering, Modeling and Applied Social Sciences, Federal University of ABC (UFABC), Santo Andre 09210-580, Brazil

**Keywords:** lignin, surface energy, wettability, practical adhesion

## Abstract

Polypropylene (PP) is a multifunctional and widely applied polymer. Nevertheless, its low energy surface and poor adhesion are well-known and might impair some prospective applications. Aiming to overcome these limitations, PP composites can be applied as a tool to enhance PP surface energy and then increase its practical adhesion. In this work, Kraft lignin (KL) was chemically modified and blended with PP. In short, KL was hydroxypropylated and further reacted with acetic anhydride (A-oxi-KL) or maleic anhydride (M-oxi-KL). Lignin modifications were confirmed by Fourier transform infrared spectroscopy (FTIR), differential scanning calorimetry (DSC), and thermogravimetric analysis (TGA). PP-composites with different lignin contents, as well as pristine PP, were characterized in terms of their thermal behavior, morphology, surface energy, and practical adhesion by DSC, scanning electron microscopy (SEM), contact angle measurement, and peeling tests, respectively. Lignin incorporation did not affect the PP degree of crystallization. The lignin modifications led to a better compatibility with the PP matrix and surface energies up to 86% higher than neat PP. Increases of up to 66% in the peel strength were verified. Composites with M-oxi-KL showed the best adhesion performance, confirming the lignin functionalization is an efficient approach to improve the practical adhesion of PP films.

## 1. Introduction

Polypropylene (PP) is a multifunctional and low-cost polymer widely used in industrial applications. Nevertheless, it presents low surface energy because of its non-polar chemically stable structure, which leads to adhesion hindrance, such as coating failures or flexible laminated layers delamination. Polymer blending can be used as a strategy to alter the wettability of PP and increase the strength of PP adhesive joints. The combination of PP with renewable resource materials has been intensively investigated as a promising approach to reduce petroleum-based dependency, hence collaborating to a reduced environmental impact. The wettability and surface roughness of polypropylene can be improved when filled with wood flour [1]. Cellulose, potato starch, and chitosan were also reacted with PP for membranes application, and the new materials changed their hydrophobicity, besides mechanical properties improvements [2].

Another strategy arises from a remarkable sustainable raw material that has been successfully applied as high-added-value for polymer blends and composites. Lignin, the second most abundant biopolymer on earth, can be found in all vascular plants and is an amorphous phenolic polymer, consisting of three main phenylpropane units (guaiacyl, synrigyl, and *p*-hidroxyphenyl) [3], that must be isolated from the other components, cellulose and hemicellulose, to be used as a technical grade [4]. Among the extraction methods, Kraft pulping, from pulp and paper industry, is the predominant process for technical lignin obtention, which is then commonly referred to as Kraft lignin [5]. In general, lignin has several advantages to be used. It is low cost, widely available, eco-friendly, non-edible, and compostable material. Its structure is composed of many different functional moieties, including aromatic rings, aliphatic and phenolic hydroxyls, carboxyls, and methoxy groups. These groups confer attractive properties, such as high mechanical stiffness, antioxidant behavior, UV absorption, thermal stability, and hydrophilization character [5,6,7]. The use of lignin in the PP matrix has been widely reported in the literature. Its hindered phenolic structure confers thermal oxidative resistance and has been a promising green substituent to synthetic stabilizers [8,9]. Mechanical properties and thermal stability were also improved by adding lignin [10,11,12].

Although encouraging results have been reported, the incorporation of lignin in a PP-matrix is not a straightforward task due to the poor compatibility that severely compromises the mechanical properties. Lignin presents difficulties in the thermoplastic molding process because of its indefinite thermal transition temperature [13]. The modification of lignin can overcome this limitation. According to Hui Li et al. [14], lignin modification is classified by two main approaches: i. Fragmentation or depolymerization into reduced molecular weight and highly functional products for further utilization as a reagent, ii. Chemical modification to introduce new reactive sites in order to optimize its structure for a specific application or desired property. Moreover, lignin chemical modification can improve its dispersion in hydrophobic polymer matrices by lowering its glass-transition temperature, which can reduce the final material brittleness while achieving higher surface energy and adhesion [7].

In this work, modified Kraft lignin was applied for the first time as an adhesion promoter for a PP matrix. The lignin was first hydroxypropylated and further esterified via two different routes in order to reduce lignin’s T_g_, thus promoting thermal processing, and enhancing the compatibility between it and the PP matrix. The chemical modifications were confirmed by Fourier transform infrared spectroscopy-attenuated total reflectance (FTIR-ATR), differential scanning calorimetry (DSC), and thermogravimetric analysis (TGA). The variation in wettability and surface energy of the PP-lignin composites were investigated. Additionally, the effect of modified Kraft lignin on the thermal properties of the composites was also explored. Finally, flexible laminated films of PP and PP-lignin composites were formed by bonding to aluminized biaxially oriented polypropylene (BOPP), and the practical adhesion was evaluated by the peeling test. The chemical modification showed a positive effect on PP surface energy and proved to be a good strategy for increasing the compatibility between PP and lignin and promoting the adhesion of this polymer.

## 2. Materials and Methods

### 2.1. Materials

Isotactic polypropylene (iPP) was supplied by Braskem (Triunfo/Brazil), with a density of 0.905 g/cm^3^ and labeled as H503HS. Kraft lignin from eucalyptus (hardwood) was kindly supplied by Suzano Papel e Celulose (Limeira/Brazil). According to the supplier, KL has pH 8.1, 92.5% solid content, and 10% ash content. The employed adhesive was a bicomponent polyurethane (PU) from Loctite, composed of the Liofol LA 9526 CP-22 adhesive and its methylene diphenyl diisocyanate (MDI) LA 6145. Propylene oxide, acetic anhydride, maleic anhydride, acetone, diiodomethane, and potassium hydroxide were purchased from Sigma-Aldrich (São Paulo, Brazil) and used as received.

### 2.2. Modification of KL

**Figure 1:**
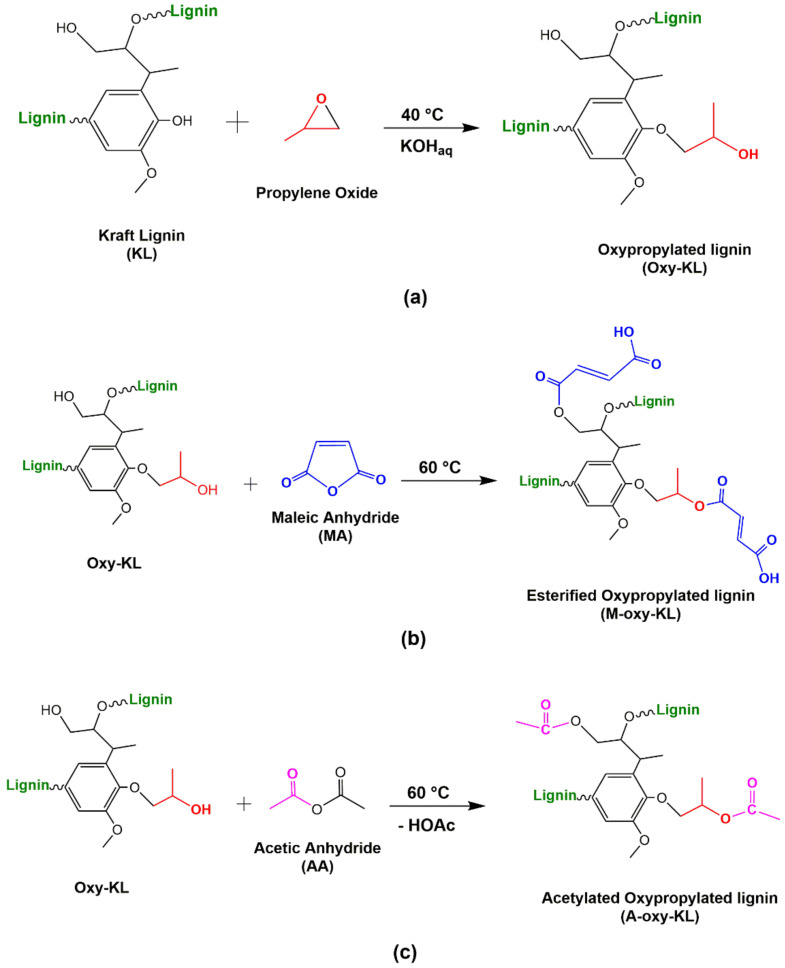
Chemical reactions employed in the lignin modification: (**a**) Hydroxypropylation with propylene oxide; (**b**) Esterification with maleic anhydride; and (**c**) Acetylation with acetic anhydride.

First, KL was hydroxypropylated, aiming to convert aromatic OH groups into aliphatic ones and to increase lignin reactivity, following the methodology presented by Garcia et al. [15]. The hydroxypropylation reaction is represented in Figure 1a. Initially, 60 g of KL were solubilized in a KOH solution (2.5 M). After homogenization, 60 mL of propylene oxide (PO) were dropwise added to the solution. The mixture was kept at constant pH (11) and under stirring at 40 °C for 1 h. Afterward, the mixture was cooled to room temperature and rested for 24 h. At last, hydroxypropylated lignin was precipitated at pH 2.5 using an HCl solution. The precipitated solid was vacuum-filtrated and washed with deionized water five times. Once hydroxypropylated, the modified KL (Oxy-KL) was reacted with maleic anhydride (MA) or acetic anhydride (AA). The esterification reaction with MA, which is presented in Figure 1b, was carried out according to [16]. The Oxy-KL was immersed in a 10% solution of MA in acetone with a ratio of 1:20 (*w*/*v*). Afterward, it was heated at reflux temperature (60 ± 2 °C) for 7 h under a magnetic stirrer in a reactor vessel. At the end of the reaction, the reactive mixture was evaporated to remove the excess acetone. The product was then vacuum-filtered and washed five times with distilled water. On the other hand, the acetylation with AA of Oxy-KL (see Figure 1c) was based on Monteil-Rivera et al. [17]. A total of 50 mg Oxy-KL was solubilized in 100 mL of AA. The mixture was kept at 60 °C, under magnetic stirring for 24 h. After this time, the mixture was then poured into distilled water (500 mL) and stirred for 2 h. The precipitated solid was vacuum filtered, washed five times with distilled water, and dried at 60 °C for 24 h.

### 2.3. Lignins Characterization

#### 2.3.1. Fourier Transform Infrared Spectroscopy-Attenuated Total Reflectance (FTIR-ATR)

Fourier transform infrared spectroscopy-attenuated total reflectance (FTIR-ATR) was performed on a Spectrum Two (Perkin Elmer, Waltham, MA, USA) instrument to investigate differences between the chemical state of modified and pristine lignin. Spectra were recorded between 3800 and 600 cm^−1^ with 32 scans and a resolution of 4 cm^−1^ at room temperature in air.

#### 2.3.2. Thermogravimetric Analysis 

TGA analyses were performed on a Mettler Toledo TGA. Ca. 8 mg of each sample was weighed and then placed onto the balance of the instrument. The samples were heated from 40 to 550 °C at a heating rate of 10 °C min^−1^ in a nitrogen atmosphere.

#### 2.3.3. Differential Scanning Calorimetry (DSC)

DSC analysis was carried out on Mettler Toledo DSC1 Star equipment. Approximately 8 mg of each sample was weighed in an alumina pinhole hermetic lid pan and then placed onto the DSC cavity. The samples were heated from 25 to 200 °C at 10 °C min^−1^, and then cooled down to 25 °C at the same rate to erase the thermal history. Then, the samples were reheated up to 200 °C at 10 °C min^−1^ and the heat flow was recorded for analysis.

### 2.4. Composites Preparation

First, samples of PP and KL and its derivatives (A-oxy-KL and M-oxy-KL) were oven-dried at 70 °C for 24 h. PP-composites, as well pristine PP, were prepared in a twin-screw extruder (Coperion, Stuttgart, Germany), by adding KL, A-oxy-KL, and M-oxy-KL to PP in different concentrations (1.0, 2.5, and 5.0 wt%), as presented in Table 1. The mixtures were carried out separately using a screw speed of 450 rpm, between 160 and 210 °C (from the feed to die) in nine-barrel temperature zones for 3 min. The extruder had a screw diameter of 20 mm and a length-to-diameter ratio L/D = 40. Then, from the resulting extruded lignin-PP granules, films about 0.3 mm thick were obtained by compression molding at 200 °C for 3 min at 4 kgf.cm^−2^ pressure for further peeling tests.

### 2.5. Composites Characterization

#### 2.5.1. Differential Scanning Calorimetry (DSC)

DSC analysis was carried out on Mettler Toledo DSC1 Star equipment. Approximately 8 mg of each composite was weighed in an alumina pinhole hermetic lid pan and then placed onto the DSC cavity. The samples were heated from 25 to 200 °C at 10 °C min^−1^, and then cooled down to −25 °C at the same rate. Then, the samples were reheated up to 200 °C at 10 °C min^−1^, and the heat flow was recorded for analysis. The glass transition temperature (T_g_) and the melting temperature (T_m_) were obtained from the 2nd heating scan, while the crystallization temperature (T_c_) was obtained from the cooling scan. The effect of lignin inclusion on the morphology of the composites was also investigated by means of the crystallization degree that was calculated from Equation (1):

(1) $$\chi \;\left(\%\right)=\frac{\Delta H_{m}}{\Delta H_{m}^{0}}\times \frac{1}{f}\times 100$$

where $\chi \;\left(\%\right)$ is the crystallization degree, $\Delta H_{m}$ is the enthalpy of fusion of the several composites as well as pristine PP, $\Delta H_{m}^{0}$ is the enthalpy of fusion of 100% crystalline PP (207 J/g) [18], and f  is the PP fraction of the composite’s composition.

#### 2.5.2. Scanning Electron Microscopy (SEM)

Specimens were manually cut for SEM analysis, aiming to investigate the cross-sectional morphology and to determine whether phase separation occurred between lignins and PP. For this purpose, samples with the highest lignin amount (5 wt%) were used. Micrographs were obtained on a Jeol 6460LV scanning electron microscope with 25 kV of accelerating voltage. The specimens were placed on the SEM sample holder and coated with a thin film of gold (~10 nm).

#### 2.5.3. Surface Energy

The solid surface free energy (γ*_S_*) was calculated by Young’s equation for the equilibrium condition at a solid–liquid interface (Equation (2)), using the contact angle (θ) of a liquid with known surface tension and the solid–liquid interfacial energy (γ*_SL_*).

(2) $$\gamma_{L}\cos\theta =\gamma_{S}\;–\gamma_{SL}$$

We used Fowkes model (Equation (3)) for the interfacial free energy between two phases to estimate γ*_SL_*, by combining both polar and dispersive interactions between the solid and liquid surfaces. In this equation, $\gamma_{s}^{d}$ and $\gamma_{s}^{p}$ are the dispersive and polar components of the surface free energy, respectively, and $\gamma_{l}^{d}$ and $\gamma_{l}^{p}$ are the dispersive and polar components of the liquid surface tension, respectively.

(3) $$\gamma_{SL}=\gamma_{S}+\gamma_{L}-2\sqrt{\gamma_{S}^{d}\gamma_{L}^{d}}-2\sqrt{\gamma_{S}^{p}\gamma_{L}^{p}}\;$$

Merging both Folke’s (Equation (2)) and Young’s (Equation (1)) equations, one can calculate $\gamma_{s}^{d}$ and $\gamma_{s}^{p}$ by obtaining the contact angle between the surface and at least two different liquids with known surface tension components.

In this study, the surface energies were determined from contact angle measurements using both distilled water and diiodomethane as liquids. The contact angle was measured by the sessile drop method with a Contact Angle Analyzer (Phoenix 300). The test drop volume was 0.015 µL, and the measurement was repeated five times for each liquid. The surface tension components of each liquid are shown in Table 2.

#### 2.5.4. Practical Adhesion

##### Lamination Process

The lamination process was performed manually according to the methodology employed by Tavares et al. [19]. PP and PP-lignin films were cut into 14 × 28 cm segments using a template. PU adhesive, which was a stoichiometric mixture of diisocyanate and biopolyol, was dissolved in ethyl acetate at a mass proportion of 50%. Polymeric films were immobilized on a flat surface for adhesive application. PU adhesive (2 mL) was applied onto the immobilized film using a 10 μm spiral lamination roller, without interruption, at constant speed and pressure. PP and PP-lignin films coated with PU were submitted to heating for ethyl acetate releasing (15 min at 50 °C). Then, an aluminized bioriented polypropylene (BOPP) layer was placed over the dried PU adhesive. The spiral lamination roller was applied again to remove trapped air bubbles. Finally, laminated sheets composed of BOPP/PU/PP and BOPP/PU/PP-lignin were obtained and cured at room temperature for seven days. After curing, the laminates were cut into samples with dimensions of 2.54 × 10 cm for peeling tests.

##### T-Peel Test

The average peel strength of BOPP/PU/PP and BOPP/PU/PP-lignin laminates was evaluated by the T-peel test using Instron 3369 universal testing. The peel tests were performed by pulling a 50-mm length of the PP from the BOPP film at an angle of 90° between the films and the direction of applied force, according to the ASTM Standard F904-98(2008) method. The tests were carried out at a crosshead speed of 0.280 m/min at room temperature. Five specimens were tested for each condition.

## 3. Results and Discussion

### 3.1. Lignins Modification

#### 3.1.1. FTI-ATR

**Figure 2:**
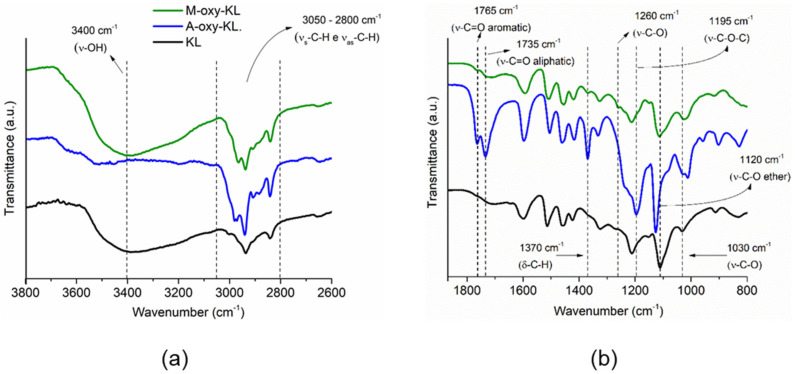
Fourier-transform infrared (FTIR) spectra of KL, A-oxy-KL and M-oxy-KL. (**a**) 3800–2600; (**b**) 1800–800.

FTIR spectra of KL, M-oxy-KL, and A-oxy-KL are shown in Figure 2 and were normalized with respect to the peak at 1514 cm^−1^ (C=C aromatic vibration) [20]. Comparing the spectra, some remarkable differences confirmed the successful KL hydroxypropylation: (i) the increase in the peak intensities and the emergence of new peaks in the wavenumber region between 3000 and 2800 cm^−1^, which are related to asymmetric and symmetrical stretching of CH_2_ and CH_3_ groups [11]; (ii) the increase in the intensity of the peak around 1370 cm^−1^, which is associated with the bending of CH_2_ and CH_3_ groups [21]; and (iii) the increase in the peak intensity at 1120 cm^−1^, which is assigned to stretching of C-O bonds of the ether groups [22].

The KL spectrum revealed a broad band centered at 3400 cm^−1^, assigned to OH groups stretching [23], which was almost completely consumed after the reaction with acetic anhydride, as observed in the A-oxy-KL spectra. Two peaks appeared at 1765 and 1735 cm^−1^, assigned to carbonyls from aromatic and aliphatic esters groups, respectively [24]. In addition, a strong peak at 1195 cm^−1^, which is related to the stretching of C-O-C bond in aromatic acetyl groups [25], was only observed in the A-oxy-KL spectra. This evidence confirmed KL acetylation. On the other hand, KL esterification via reaction with maleic anhydride was confirmed by the following evidence observed when KL and M-oxy-KL spectra are compared: (i) the emergence of a weak peak centered at 1765 cm^−1^ (C=O stretching of aromatic ester); (ii) the increase in the intensity of the peak around 1260 cm^−1^ and the increase in intensity and width of the peak centered at 1030 cm^−1^, both associated with the stretching of C-O bonds of ester groups [8].

#### 3.1.2. Lignin Thermal Analysis

**Figure 3:**
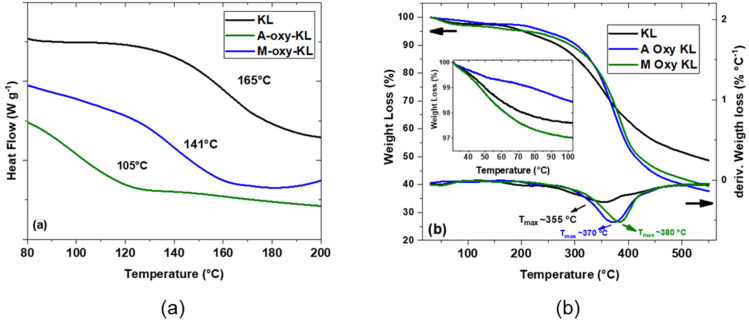
(**a**) Heat flow evolution with temperature of pristine lignin (KL) and its derivatives (A-oxy-KL and M-oxy-KL) (**b**) Weight loss (%) (left axis) and derivative of weight loss (right axis) curves of pristine lignin (KL) and its derivatives (A-oxy-KL and M-oxy-KL). Insert: zoom on the 40 to 100 °C range to highlight the differences in moisture loss.

Pristine lignin and its derivatives’ thermal behavior was assessed via DSC analysis to confirm the occurrence of chemical modification. Figure 3a shows the heat flow evolution with temperature for the pristine KL and its derivatives. The glass transition temperature (T_g_) was assumed as the peak temperature of the derivative of the heat flow (the curves were omitted for the sake of simplicity). The T_g_ of KL was estimated at 170 °C, which agrees with the literature that has reported the T_g_ of lignin ranging from 80 °C to 180 °C, depending on the botanical source and/or extraction method [26]. After chemical modification, there is an evident decrease in T_g_ that reaches 105 °C for A-oxy-KL and 141 °C for M-oxy-KL. The decrease in the lignin T_g_ upon chemical modification was expected and has been demonstrated in several works that employed different reaction routes [16,20,27,28,29]. This trend is mainly explained by two mechanisms. The first one is related to the presence of large lateral groups that increase the molecule’s free volume hence decreasing the T_g_ [30,31]. The second is associated with the effect of chemical modification on intermolecular interactions. After KL hydroxypropylation, the −OH moieties are replaced by large OH-terminated aliphatic branches. After further acetylation with acetic anhydride, these hydroxyls are replaced by acetyl groups (−OCH_3_), as evidenced by FTIR-ATR analysis. Clearly, acetyl groups possess a lower polarity that weakens the hydrogen bonding network, which severely impacts lignin macromolecular dynamics and, consequently, its T_g_ [32,33]. On the other hand, maleic anhydride-modified lignin has a carboxyl ending group that presents an intermediate polarity between −OH and −OCH_3_, and therefore, a less pronounced effect on the intermolecular interaction, yielding a more modest decrease of the T_g_ compared to pristine lignin. 

The thermograms of KL and chemically modified KL are presented in Figure 3b (left axis). Around 100 °C there is a small weight loss for all samples (lower than 5%) that is attributed to water evaporation and is commonly seen during lignin heating scans. Among the three samples, A-oxy-KL presented the lower weight loss in this stage (Figure 3b inserts). This is a consequence of lignin hydrophobization upon acetylation of −OH moieties in the lignin structure, and a similar effect has been seen elsewhere [34,35]. Around 250 °C a more abrupt decline in the weight percentage can be seen in all the samples as an indication of the main degradation step that occurs between 250 and 500 °C and is related to the decomposition of alkyl-ether linkages, dehydration, and decarboxylation reactions [36,37]. Lignin chemical modification slightly increased the thermo-resistance compared to pristine lignin. This is mirrored by the lower maximum degradation temperature of the latter (350 °C) in comparison to the chemically modified counterparts (370 and 380 °C for A-oxy-KL and M-oxy-KL, respectively), as evidenced by the derivative of the weight loss (Figure 3b, right axis).

The char mass residue at 550 °C is found to decrease with lignin chemical modification compared to KL. The char residue after lignin pyrolysis is associated with the high thermal resistance of the aromatic backbone in its chemical structure [38,39]. Upon chemical modification by hydroxypropylation followed by acetylation, large aliphatic branches were included in the lignin structure, which reduces the char residue as confirmed in previous works that attempted a similar approach [40,41]. Therefore, both thermal analyses provide strong evidence of the successful chemical modification of KL by hydroxypropylation followed by esterification with acetic anhydride and maleic anhydride.

### 3.2. Composites Characterization

#### 3.2.1. DSC

**Figure 4:**
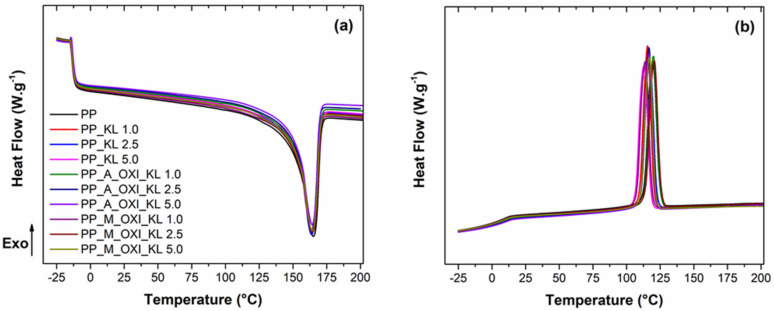
Heat flow evolution with temperature for pristine PP and PP/lignin and its derivative composites during (**a**) the second heating scan, featuring the glass transition and the melting peak, and (**b**) the cooling scan, featuring the crystallization peak.

DSC analysis was carried out to evaluate the effects of lignin incorporation on the thermal events of the PP matrix. Additionally, these results might also provide some information regarding the composite’s microstructure by means of the degree of crystallization ($\chi \left(\%\right)$), calculated according to Equation (3). Table 3 summarizes the main thermal events as well the crystallization degrees, while the thermograms are shown in Figure 4.

In general, the inclusion of lignin and its derivatives did not significantly affect the T_g_ and T_m_ values and shape when compared to the neat PP. Moreover, no clear signal of the lignin phase T_g_ can be seen in any of the thermograms. Although astonishing, similar results have been reported in several works with PP and other polymers [42], including a previous one from our research group [7], and is mainly explained by the stiff lignin molecules that hinder T_g_ measurements when blended or diluted in other polymers [43]. Additionally, there is possibly an overlap of the T_g_ of the lignin phase with the large endothermic melting peak of PP that might also impair the observation of the two events.

On the other hand, the crystallization peak observed during the cooling scan shows a dependence on the lignin inclusion. In general, the T_C_ slightly decreases, and the crystallization peak width increases with the inclusion of lignin. This tendency might be associated with a slower crystallization process [30,43], due to the overall lower mobility of the PP chains upon lignin inclusion. In spite of the somewhat slower crystallization, the inclusion of lignin did not affect the final crystallization degree, as can be seen in the last column of Table 3. Pristine PP showed a crystallization degree of 9.86%, while the inclusion of lignin and its derivatives causes no meaningful change in this value. This is an interesting result, as it suggests that there is no significant alteration of the morphology of the composites that could impact the adhesion behavior in the peel strength test, which is discussed in Section 3.2.4.

#### 3.2.2. SEM

**Figure 5:**
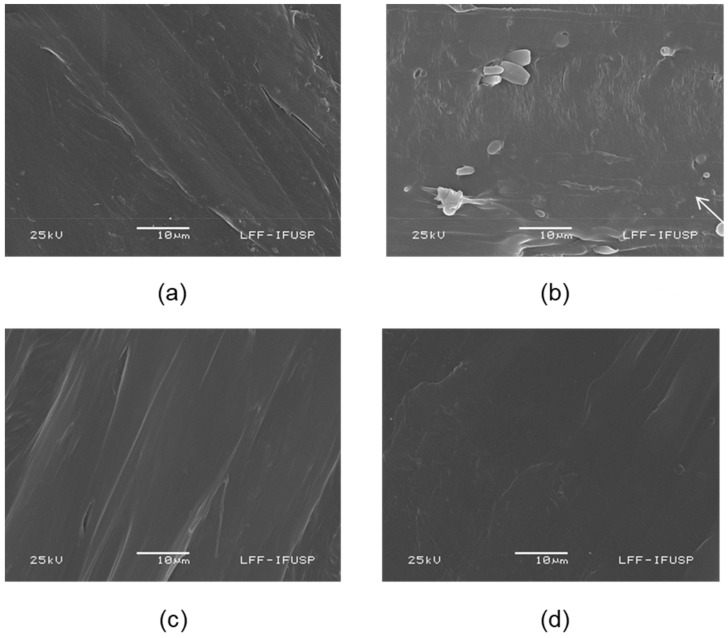
Cross-section scanning electron micrographs of pristine PP and PP composites contaning 5 wt% of lignin: (**a**) pristine PP; (**b**) PP/KL composite; (**c**) PP/A-Oxy-KL; (**d**) PP/M-Oxy-KL.

Figure 5 presents the micrographs of pristine PP and PP composites with 5 wt% of lignin. First, the SEM analysis revealed the overall presence of patterns in the cut cross-section of the KL-containing composite, represented in Figure 5b (white arrows). This pattern is quite similar to lignin shape, previously reported elsewhere in KL micrographs [7], and might be related to KL aggregation or phase separation. It is well known that PP and unmodified Kraft lignin are immiscible due to the different natures of both materials, which results in weak dispersion and, consequently, in a heterogeneous morphology with lignin aggregates dispersed in a continuous PP phase [44]. In the literature, besides PP and KL incompatibility, phase separation is usually reported for composites with higher lignin contents (>10 wt%) [45,46]. However, the previously reported results were obtained from different types of PP, lignin, or processing. On the other hand, composites here investigated with modified lignin exhibited no patterns, revealing the absence of lignin aggregation and indicating a better interaction between PP and modified lignins. This result reveals that both chemical modifications improved the compatibility between lignin and the PP matrix. Lignin modification enhances its dispersion into PP due to the inclusion of aliphatic branches and the weakening of intermolecular interactions between lignin molecules (as discussed in the context with DSC analysis). Furthermore, the weakening of intermolecular interactions decreases the tendency of agglomeration [47]. Therefore, a more homogeneous and uniform morphology is expected.

#### 3.2.3. Surface Energy

The contact angles obtained for each substrate using either deionized water or diiodomethane are shown in Table 4. The contact angle measured for water on the pristine PP film was significantly higher than those obtained for PP films with any percentage of lignin, even for PP-KL composites, in which agglomeration and poor dispersion of lignin were observed by SEM analysis. This is likely because the added lignin samples have hydroxyl, acetyl, and carboxyl groups that increase the film’s overall polarity and thus its hydrophilicity.

**Figure 6:**
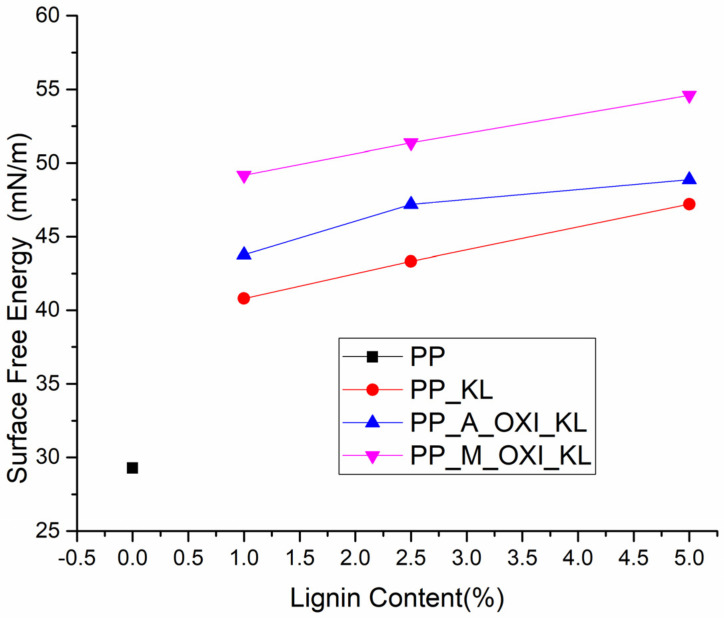
Calculated surface energies of the PP and PP-lignin films. The surface free energy of the PP film without any lignin is represented as a black line for all lignin content % for comparison purposes.

The calculated surface energy of neat PP, Figure 6, was 86% lower than the best result for the PP-lignin composites verified for the composite with 5 wt% of M_Oxi_KL (54.57 and 108.55 mN/m, respectively). Samples containing 5 wt% of KL, A-Oxi_KL, and M_Oxi_KL showed an increase of the surface free energy up to 15.7%, 11.7%, and 11.0% in comparison to their 1 wt% counterparts, respectively. This can be related to the surface wettability in which a higher surface energy value corresponds to a stronger interaction between liquid molecules and the films produced. The best results verified for modified lignin are associated with a better dispersion and compatibility displayed by its respective composites.

#### 3.2.4. T-Peel Test

**Figure 7:**
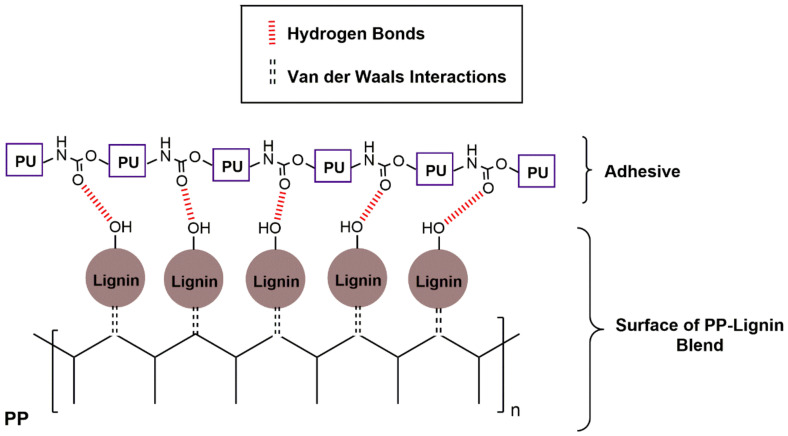
Proposed adhesion mechanism between PP-lignin composites and the adhesive.

The T-peel test results are shown in Table 5. All the samples displayed predominantly adhesive failure. The average peel strength of the laminates increased with the lignin incorporation, regardless of the lignin type. Increased lignin concentration resulted in an improvement of practical adhesion of the laminated BOPP/PP films. These results converge with contact angle measurements since the addition of all types of lignin led to increases in PP surface energy due to the presence of polar groups, especially hydroxyls, in the lignin structure. Since the degree of crystallization of PP was not affected by lignin incorporation, as indicated by DSC results, the improvement in adhesion performance might, in principle, be attributed to this change in surface energies. Surface energy is a critical factor in predicting adhesive joint performance since it is related to the formation of adhesion bonds [45,48]. Materials with higher surface energy tend to exhibit stronger attractive forces than those with low surface energy, providing higher wettability, allowing a better spread of the adhesive and, consequently, leading to a higher adhesion. Figure 7 shows the possible adhesion mechanism between PP-lignin composites and PU adhesive that could clarify the improvements in the laminates’ average peel strength. This mechanism is based on the formation of hydrogen bonds between the lignin hydroxyl groups and adhesive polar groups. The adhesive failure reinforces the occurrence of this mechanism.

Similar results were reported by de Sousa et al. [6], which investigated the effect of the incorporation of unmodified acid (AKL) and basic (BKL) Kraft lignin on the adhesion properties of PPs. The authors verified increases of up to 45% and 20% in the PP peel strength compared to a neat PP by the addition of AKL and BKL, respectively. However, unlike observed in the present work, when the BKL content rose, the peel strength gradually decreased. On the other hand, AKL-containing PP composites exhibited a non-linear trend regarding the increase in lignin content.

Among all lignin types, the hydroxypropylated lignin further reacted with maleic anhydride (M_Oxi_KL) provided the highest practical adhesion. Laminates containing PP blended with 5 wt% of M_Oxi_KL showed a peel strength 66% higher than neat PP. For the same concentration, the incorporation of unmodified lignin (KL) and hydroxypropylated lignin acetylated (A_Oxi_KL) led to increases of 13% and 46% in the peel strength, respectively. Furthermore, laminates containing only 1 wt% of M_Oxi_KL exhibited higher peel strength than all those containing KL and A_Oxi_KL, even at 5 wt% loading. The results revealed a positive effect of lignin chemical modification on the practical adhesion of the laminates. This positive effect can be associated with better compatibilization between PP and modified lignin [46,47], confirmed previously by SEM analysis. Consequently, surface and mechanical properties are improved [49,50]. Both properties contributed to the adhesion improvement of PP/lignin composites in BOPP/PU/PP-lignin laminates. Furthermore, in addition to the better compatibility, lignin modification led to an increase in molecular mobility (see DSC results), which could make the diffusion of modified lignin to the surface easier, thus improving practical adherence.

## 4. Conclusions

Kraft lignin was chemically modified and further incorporated into PP for surface energy increase. Hydroxypropylation followed by esterification with maleic anhydride and acetylation with acetic anhydride reduced the Kraft lignin T_g_ from 165 to 141 °C and 105 °C, respectively, and improved the compatibility between Kraft lignin and the PP matrix. In general, lignin incorporation did not affect the degree of crystallization and, consequently, the morphology of PP decreased the contact angle of PP composites, and thus increased their surface energy. M_Oxi_KL-containing composites developed higher surface energy compared with their counterpart. The strength of the adhesively bonded joints, or practical adhesion, followed the same trend of energy surface results: lignin addition increased the laminate’s practical adhesion, in which M_Oxi_KL-containing composites enhanced the laminate peel strength to 45.15 N/m, which represents an increase of 66% in comparison to pristine PP samples. Indeed, Kraft lignin modification emerges as a promising method for the development of a green adhesion promoter.

## Figures and Tables

**Table 1:** Sample nomenclatures and compositions.

Name	PP (%)	KL (%)	A-oxy-KL (%)	M-oxy-KL (%)
PP	100	-	-	-
PP_KL_1	99	1	-	-
PP_KL_2.5	97.5	2.5	-	-
PP_KL_5	95	5.0	-	-
PP_A_ Oxi_KL_1	99	-	1	-
PP_A_Oxi_KL_2.5	97.5	-	2.5	-
PP_A_Oxi_KL_5	95	-	5.0	-
PP_M_Oxi_KL_1	99	-	-	1
PP_M_Oxi_KL_2.5	97.5	-	-	2.5
PP_M_Oxi_KL_5	95	-	-	5.0

**Table 2:** Surface tension energy of the test liquids [7].

Surface Tension	Water	Diiodomethane
Total (mN/m)	72.8	50.8
Polar (mN/m)	51.0	0
Disperse (mN/m)	21.8	50.8

**Table 3:** Peak temperatures (°C) of the main thermal events observed during the cooling and second heating scan. The final column shows the crystallinity degree (*χ* %), obtained from the melting peak of the second heating scan.

Sample	T_g_ (°C)	T_c_ (°C)	T_m_ (°C)	*χ* (%)
PP	−13.24	130.76	165.1	9.86
PP_KL_1	−12.94	126.53	164.1	9.73
PP_KL_2.5	−13.04	128.50	163.8	9.89
PP_KL_5	−13.13	122.91	164.6	9.59
PP_A_ Oxy_KL_1	−12.35	130.86	164.2	10.16
PP_A_Oxy_KL_2.5	−12.45	126.22	163.5	9.71
PP_A_Oxy_KL_5	−12.15	126.22	164.0	9.96
PP_M_Oxy_KL_1	−13.13	124.10	164.2	9.54
PP_M_Oxy_KL_2.5	−13.04	131.81	164.3	10.02
PP_M_Oxy_KL_5	−12.84	126.62	164.2	9.78

**Table 4:** Contact angles for water and diiodomethane on PP and PP-lignin films.

Sample	Water (°)	Diiodomethane (°)
PP	78.17 ± 0.61	36.87 ± 1.89
PP_KL_1	66.12 ± 2.20	30.52 ± 2.02
PP_KL_2.5	63.25 ± 1.07	33.82 ± 0.76
PP_KL_5	60.12 ± 1.10	38.89 ± 1.32
PP_A_Oxi_KL_1	63.21 ± 0.31	30.15 ± 2.07
PP_A_Oxi_KL_2.5	60.02 ± 0.80	35.09 ± 1.87
PP_A_Oxi_KL_5	58.36 ± 2.01	33.95 ± 1.02
PP_M_Oxi_KL_1	58.02 ± 1.87	32.71 ± 2.89
PP_M_Oxi_KL_2.5	55.89 ± 2.10	31.64 ± 2.01
PP_M_Oxi_KL_5	52.61 ± 2.85	26.78 ± 2.89

**Table 5:** Average peel strength of BOPP/PP and BOPP/PP-Lignin adhesively bonded joints.

Sample	Average Peel Strength (N/m)
PP	27.20 ± 0.84
PP_KL_1	28.72 ± 0.32
PP_KL_2.5	29.15 ± 0.55
PP_KL_5	30.78 ± 0.43
PP_A_ Oxi_KL_1	35.26 ± 0.66
PP_A_Oxi_KL_2.5	37.68 ± 0.91
PP_A_Oxi_KL_5	39.58 ± 0.13
PP_M_Oxi_KL_1	39.74 ± 0.89
PP_M_Oxi_KL_2.5	42.80 ± 1.02
PP_M_Oxi_KL_5	45.15 ± 1.28

## Data Availability

The data presented in this study are available on request from the corresponding author.
